# Pragmatic Use of Planetary Health and Nature-Based Solutions for Future Pandemics Using COVID-19 Case Scenario

**DOI:** 10.3389/fpubh.2021.620120

**Published:** 2021-05-20

**Authors:** Elena Boriani, Maurizio Aragrande, Massimo Canali, Mario V. Balzan, Muhammad Asaduzzaman

**Affiliations:** ^1^Independent Researcher, EB Consult, Hellebæk, Denmark; ^2^Department of Agricultural and Food Sciences, University of Bologna, Bologna, Italy; ^3^Malta College of Arts, Science and Technology, Institute of Applied Sciences, Paola, Malta; ^4^Faculty of Medicine, Centre for Global Health, Institute of Health and Society, University of Oslo, Oslo, Norway; ^5^Planetary Health Alliance, Boston, MA, United States

**Keywords:** COVID-19, SARS-CoV-2, Planetary health, environmental policy, systems thinking, nature based solutions, emergency preparedness, knowledge matrix

## Introduction

The coronavirus disease 2019 (COVID-19), which started in December 01, 2019, in Wuhan, China (1), is the most influential public health event at this moment. Experts are working to control this infection with several scientific measures, such as mathematical modeling, social containment, or multiple trials of new drugs and vaccines, which seem the only possible strategies to face the very wide “unknown” about the virus. The discovery of several vaccines for COVID-19 still poses problems determined by production capacity, contractual accuracy, and probably opportunistic behavior that may hinder the application of the scientific discovery. The social and economic impacts of the measures undertaken show that their application require careful evaluation of sustainability and trade-offs between acceptable health risk and societal costs. The possible effect of the current economic model of planetary resource exploitation on the emergence of pandemics and, on the other hand, the relationship between the implementation of containment measures and the institutional system at the national level, are other critical issues of the current pandemic that add complexity to the above-mentioned action framework. In fact, our view of the current pandemic is much in line with the conceptualization of Planetary Health defined by the Rockefeller Foundation-Lancet Commission on Planetary Health (2) as “the achievement of the highest attainable standard of health, well-being, and equity worldwide through judicious attention to the human systems—political, economic, and social—that shape the future of humanity and the Earth's natural systems that define the safe environmental limits within which humanity can flourish.” We also believe that nature-based solutions (NbS) are an important approach to preventing future pandemics that focuses on “transdisciplinary research into the design and implementation of solutions based on nature”(3) through linking various ecosystem management tools as positive natural resources. In this context, we argue for systems thinking and interdisciplinarity, which are described here as the key methods to address the intrinsic complexity of these Planetary Health-related problems, through the integration of different models and visions of a problem (4–6). This is not new to the holistic approaches to health, but what we propose is a way to make them simple and viable as much as possible for people involved in the solution of complex health problems.

## How Planetary Health Fits With COVID-19

To understand COVID-19 in the context of Planetary Health, we need to conceptualize the definition mentioned above (2). In line with this definition, COVID-19 is a clear demonstration of the disruption of the natural ecosystem with a massive shock to existing political, economic, and social structure globally. Several phylogenetic analyses have confirmed the linkage of SARS-CoV-2 with wildlife, especially with the severe acute respiratory syndrome-like (SARS-like) bat viruses (7) including some other intermediate host in wildlife also. Due to unplanned economic growth and the rapidly increasing global population, about 7–11 million km^2^ of forests in the world have been destroyed with a loss of half of the planet fauna, including amphibians, mammals, reptiles, birds, etc., in the last 45 years (8). Rapid urbanization, land degradation, wildlife trade, and loss of biodiversity are the root causes of frequent epidemics or pandemics originating in domestic or wild animals. The occurrence of SARS (2003) and SARS-CoV-2 (2019) in China (7) is not unusual. The infographic published with Rockefeller Foundation-Lancet Commission on Planetary Health (2) clearly depicts Planetary Health as a discipline cutting across the health sciences (Human Health) and the natural and physical sciences, such as agriculture, biodiversity conservation, ecology, environmental sciences, and urban planning, which are also closely related to the current COVID-19 crisis. In addition, these planetary aspects of the pandemic become linked to health issues such as infectious diseases, respiratory medicine, virology etc.

## Highlights of the Pragmatic Methodology

The methodology is based on two main steps, i.e., the identification of the system and its boundaries and hybridization of knowledge. System identification and boundary setting may stem from a disciplinary view of a problem under the condition that an iterative process is started to include more disciplines to widen the vision of the problem and the understanding of its complexity. This comes by recognizing the limits of each discipline and addressing questions that enable us to shed light on how other disciplines may contribute to expand our knowledge (i.e., what more should be known about the problem, how we can go about answering this question, and who has the knowledge). Awareness of complexity progresses by identifying the building blocks of a problem and their relationships. This allows us in turn to develop a matrix of knowledge for which disciplinary knowledge is assigned roles in order to understand complexity in an inter- or trans-disciplinary framework, i.e., by identifying (i) the domain of expertise (what aspects are taken into account by the knowledge): (ii) the advancement in knowledge it creates (what useful knowledge can be obtained or expected which can contribute to the understanding of the problem); (iii) the method(s) to obtain it (9).

## Building Blocks (BB), Systems Thinking, and Planetary Health

**Figure 1:**
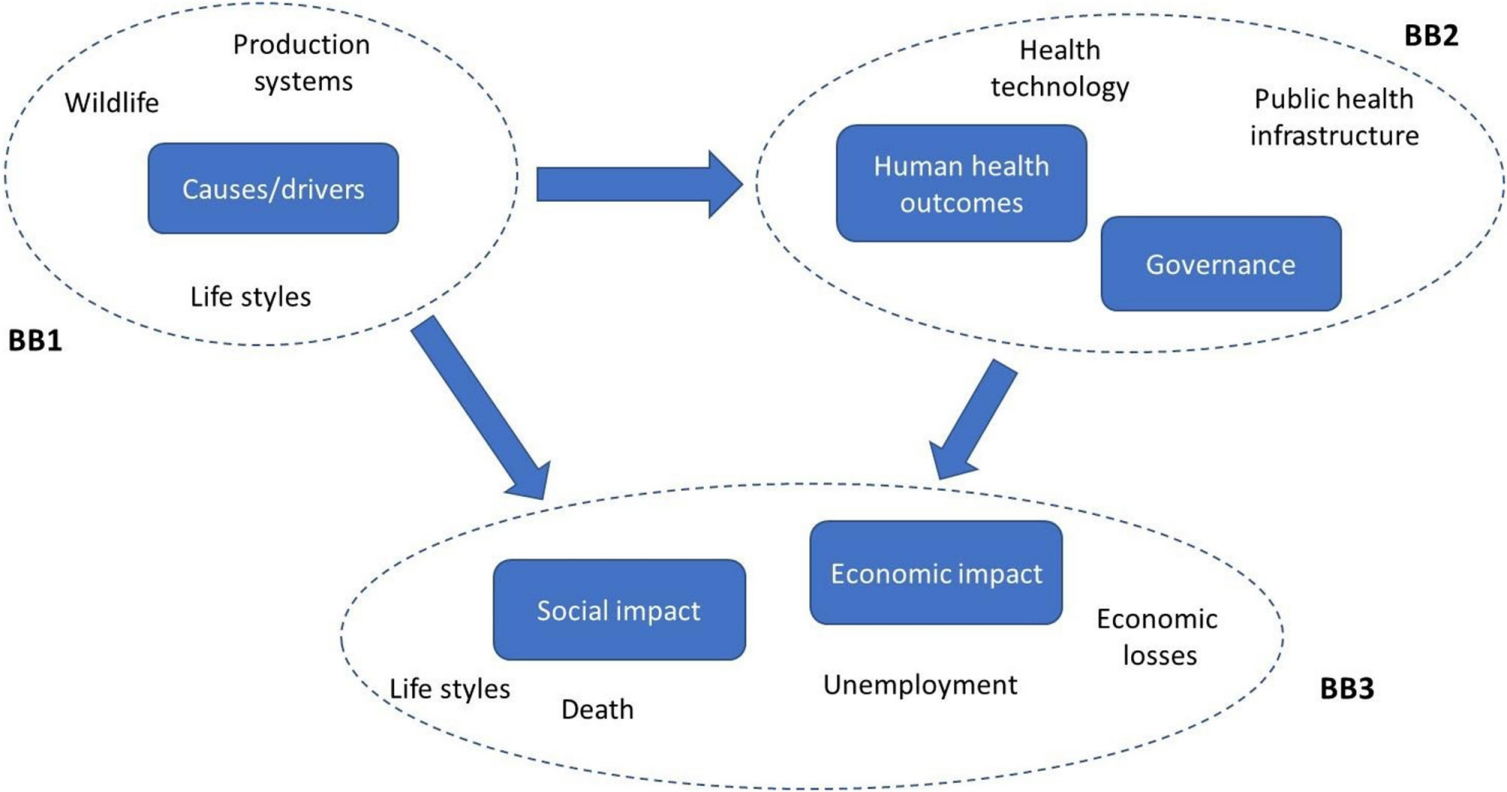
Building blocks in systems thinking for COVID-19 like scenario.

The COVID-19 pandemic has revealed some common traits in the behavior of national governments and public health authorities around the world. The virus was largely unknown, and mitigation measures were based mainly on the elementary precaution of social distancing (lockdown strategy) and waiting for scientific advancement on the medical side. Lockdowns, adopted after an initial phase of general underestimation of the problem, translated into a reduction of the pandemic and in relevant economic losses. The current strategy is based on restarting economic activities in the context of the development of vaccination programs and a possible coexistence with the virus. Individual and social behaviors still play a relevant role in avoiding the restart of COVID-19 on a large scale, together with the measures aimed at the early detection of the hot spots. In this context, we find it useful to try an application of our pragmatic method, not to find out deterministic solutions but just to put forward the idea that considering pandemic complexity with different eyes can contribute to identifying effective solutions. We focus in particular on the building blocks of the system, keeping in mind that our objective is not to provide an exhaustive image of the system but to be suggestive about the method to reach it. Reasoning in a small interdisciplinary group, we identified three main building blocks of the COVID-19 problem at the current stage, as outlined in Figure 1.

BB1 refers to the understanding of the causes and drivers of pandemic. This is much related to the use of natural resources made by the population (i.e., the relationship between humans and animals in specific contexts) and the production system (i.e., the development model of the society), namely its sustainability. Once the virus hits humans, human health problems arise (BB2), showing the limits of the available knowledge/technology and health infrastructure to face the health consequences of the epidemic. BB3 focuses on the consequences of the pandemic and the lockdown for the society: severe diseases and death, social isolation, and the dramatic limitation of lifestyle, the fall in production, unemployment, and welfare losses. Each of the above-mentioned BBs is characterized by an intrinsic complexity that can be understood only using different competencies. For example, problems rising in the BB1 require that natural scientists, sociologists, and economists work together and with local communities/stakeholders to understand what situations are occurring at the interface of wildlife, society, and the economic system. At a wider level, BBs are closely linked each other. Knowledge outcomes from BB1 can determine advances in BB2, especially for human care and technology. This in turn can change pandemic governance and limit the social and economic impact. At the same time, BB1 outcomes may suggest ways to prevent the risk of zoonosis at an early stage by focusing on consumers' behavior and sustainable uses of natural resources. According to the iterative process and answering the basic questions mentioned above, building blocks can be added and/or modified, including more perspectives and points of view on the problem, thus progressing in our understanding of its complexity.

## The Planetary Health Knowledge Matrix and Its Use in Nature-Based Solutions (NBS)

In view of the considerations above, a matrix of knowledge can be formulated. Supplementary Table 1 is an initial exercise to derive the matrix of knowledge from the BBs depicted in Figure 1. Here we have limited ourselves to listing the scientific domains and expertise that appear from a preliminary assessment of the problem's complexity. The matrix should be completed in the vertical sense, by adding rows (i.e., disciplines, competent institutions, stakeholders, and social parties that have relevant knowledge about the problem) and columns (i.e., assigning to each actor of the system—scientists, institutions, and stakeholders—a specific role in producing the required knowledge and methods to obtain it). The general matrix we drafted is of course incomplete because, as mentioned above, our aim at this stage is to suggest a mode of reasoning, not to provide deterministic solutions. Just to exemplify how the matrix of knowledge works, we limit ourselves here to highlighting the role that some disciplines can play in relation to other disciplines to expand systemic knowledge. At this stage, questions are more relevant than answers. Economics is often called into question to assess the cost of the strategies to fight COVID-19 (in particular, the consequences of lockdowns). Though relevant, this role is limiting, and it just follows the traditional monodirectional causation stemming from an event (the pandemic). According to a systemic vision, we should ask what are the pandemic's economic drivers: which situations lead to overexploitation of natural resources? Which are the economic determinants of consumer behavior in relation to wildlife? Is economics able to answer these questions? What are the other disciplines that can increase systemic knowledge? These are the underlying questions that have led the authors to create Supplementary Table 1. If we limit ourselves to an elementary description of BB1 in Figure 1, it is almost intuitive to understand that natural scientists, veterinarians, sociologists, and economists should work together to understand the complex drivers of resource use, the conflict between conservation and production, and the related effects on the potential risk of pandemics. Of course, this exercise is strongly limited by the expertise of the authors, who do not cover all the disciplinary issues related to the case under discussion. On the other hand, as stated elsewhere in this paper, the objective is exactly to show the limits of disciplinary approaches and the need for more interdisciplinary collaboration.

The method we propose is aimed at easing the approach to systems thinking and inter- and trans-disciplinary thinking also in people who do not practice those methods. Further work is needed to refine the complete application of these approaches. We believe that showing the immediate advantages of the method and the real need behind it can gain followers of the method. Our experience with COVID-19 shows that, beyond emergency measures, resources must be devoted to a global, holistic vision of the problem. We are limited here to suggesting a pathway for systems thinking and interdisciplinary cooperation with simple reasoning and examples. Other dimensions of the problem should be considered. The geopolitical dimension of the pandemic and the variety of national and international institutions concerned show that finding solutions to COVID-19 should also involve the institutional and political dimensions of the system. The COVID-19 crisis hits the poor, migrant, and refugee populations to a larger extent, which leads to food insecurity and healthcare disparity (10, 11). Other more specific areas of interdisciplinary collaboration can be mentioned. For example, increased and intensified human activity in forest areas and climate change have turned bats into reservoirs of emerging and reemerging pathogens, including both RNA and DNA viruses (12). Again, the body temperature of bats is high when they fly for food or other purposes at least twice a day, and they can act as reservoirs for various deadly pathogens such as the Nipah, Ebola, Hendra, SARS-CoV-2, and Marburg viruses (13). It might be the explanation for why bat-transmitted viruses are capable of spreading in high temperatures. However, no drastic measures can be taken due to the crucial role of bats in our ecosystem, especially in pollination, seed dispersion, and insect control (14). Knowing these facts, to act accordingly, we require wildlife and ecology experts to contribute to systems thinking. Another important context is the urban green spaces (UGS) or urban natural green infrastructure (NGI), which have demonstrated a wide range of health, social, and environmental benefits (15–18). However, most of the health benefits focus on mental health, healthy aging, quality of life, perinatal health, and various chronic diseases such as cancer, diabetes, heart disease, and so on (18). We need to focus on innovative ideas to utilize such NbS as access to UGS or NGI for gaining the utmost health benefits in communicable or infectious diseases. For example, in the case of COVID-19, social distancing with access to green space would be more beneficial than social distancing alone. A recent study (19) conducted in three cities on three different continents showed a significant change in the human microbiota in the nose and skin after exposure to UGS. There is also scientific discussion ongoing to adopt the exposome-based urban public health intervention in pandemic/epidemic situations such as COVID-19, where the built-in environment indicators (e.g., NGI, UGS) are just as important as population and individual characteristics both in terms of disease causation and outcomes (20, 21). Such study findings can be the way forward to identify the role of NbS in tackling future pandemics resulting from environmental pathogens.

## Conclusion

The global pandemic of COVID-19 is still ongoing and the exact time point of its full containment cannot be anticipated yet. Though some medical solutions like vaccines have been made available, the chance of future pandemics of similar characteristics cannot be ruled out, which stresses the key role of preparedness. However, preparedness calls into question many different aspects, which are not limited to biomedical issues (from research on pathogens to health infrastructure) but also involve various actors in the wider sense. The currently adopted containment measures (e.g. social distancing, the extended lock down), their effect on production and revenue, social practices and lifestyle are showing that the effectiveness of these measures requires careful consideration of social and economic sustainability. Therefore, only the public health measures or economic or social policy are not enough to tackle such situation or its aftermath. If we are convinced about the importance of Planetary Health in emerging infections and associated pandemics, the use of a pragmatic systems thinking methodology is strongly suggested to make the specific disciplines and institutional competences work together. Based on the Planetary Health knowledge matrix, NbS can be considered as a great tool in the containment of current and future pandemics.

## Author Contributions

MAs, MAr, and EB: context, literature search, data interpretation, and writing. MC and MB: revision and table compilation. All authors approved the manuscript.

## Conflict of Interest

The authors declare that the research was conducted in the absence of any commercial or financial relationships that could be construed as a potential conflict of interest.
