# Supplementary material for: Pragmatic Use of Planetary Health and Nature-Based Solutions for Future Pandemics Using COVID-19 Case Scenario
Source: Front Public Health. 2021 May 20;9:620120. doi: 10.3389/fpubh.2021.620120 (PMC8172619; doi:10.3389/fpubh.2021.620120)
Supplement: Supplementary file 1 [file Table_1.DOCX]

**Table S1 – Example of knowledge matrix for COVID-19**

| - **Knowledge** | - **Expertise** | - **Advancement in knowledge** | - **Method** |
| --- | --- | --- | --- |
| - Public Health & Human medicine | - Public Health & Human medicine | - Identification of zoonotic diseases and their effective management | - Outbreak investigation, active case management, vaccine & drug production |
| - Veterinary medicine & Wildlife expert | - Veterinary Public Health | - Disease occurrence from bat or wildlife exposure | - Outbreak investigation, active case management, vaccine & drug production |
| - Biodiversity conservation | - Conservation policy | - Conservation of natural habitat, wildlife | - Monitor of indicators, species - Ensure habitat conservation to provide resources and conditions required by wildlife |
| - Ecology | - Nature based solution and interventions | - Ecological modeling, Ecosystem service | - Monitoring the harmonization among organism, population, community, ecosystem, and biosphere. |
| - Anthropology, sociology | - Study of the population | - Reaction from human species to measures to stop the pandemic eg confinement - Impacts of pandemic on human society and variation of these with socio-economic conditions | - Gender equality monitoring |
| - Environmental sciences | - Environmental evaluation | - Environmental dimensions of zoonotic diseases | - Monitoring animal risk assessment |
| - Health and environmental economics | - Economic evaluation | - Identification of public and private cost | - Cost analysis methods, cost-benefit analysis |
| - Migrant Health | - Risk estimation and population specific study | - Identification of language, cultural and access to healthcare barrier | - Developing migrant centered health promotion and healthcare delivery |
